# Supplementary material for: Identification of photoperiod-induced specific miRNAs in the adrenal glands of Sunite sheep (Ovis aries)
Source: Front Vet Sci. 2022 Jul 22;9:888207. doi: 10.3389/fvets.2022.888207 (PMC9354845; doi:10.3389/fvets.2022.888207)
Supplement: Supplementary file 1 [file Data_Sheet_1.ZIP › Supplementary file/miRDeep2 file.zip!/miRDeep2 file/Know_Structure/SP42A3/oar-mir-152.pdf]

miRBase precursor : oar-mir-152
Total read count : 17280
oar-miR-152 read count : 17280
remaining reads : 0

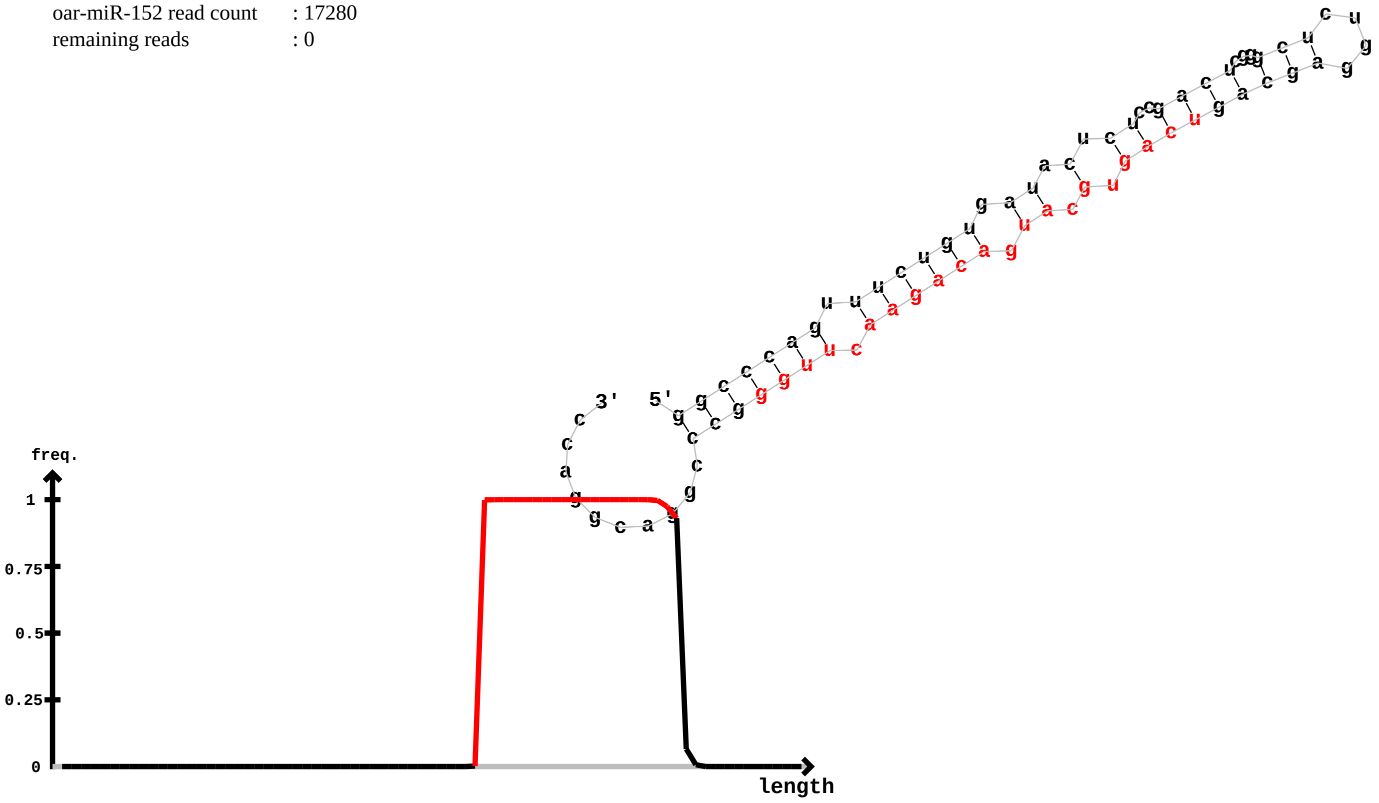


```
                                                oar-miR-152
5'- ggcccaguuucugugauacucuccgacucgggcucuggagcagucagugcaugacagaacuugggcccggacggacc   -3'    exp
    (((((((.((((((.((.(.((..((((...(((....)))))))))).).)).)))))).))))))).........    reads      mm         sample
    ..........................................gucagugcaugacagaacuug..............    6          0          seq
    ..........................................gucagugcaugacagaacuugg.............    26         0          seq
    ..........................................gucagugcaugacagaacuuggg............    1          0          seq
    ...........................................ucagugcaugacagaac.................    2          0          seq
    ...........................................ucagugcauCacagaac.................    3          1          seq
    ...........................................ucagugcaugacagaacu................    35         0          seq
    ...........................................ucagugcauCacagaacu................    3          1          seq
    ...........................................ucagugcauCacagaacuu...............    16         1          seq
    ...........................................ucagugcaugacagaacuu...............    386        0          seq
    ...........................................ucagugAaugacagaacuug..............    3          1          seq
    ...........................................ucagugcaugacagaacuug..............    746        0          seq
    ...........................................ucagugcaugacagaacuugg.............    14907      0          seq
    ...........................................ucagugcaugacagaacuuggg............    1014       0          seq
    ...........................................ucagugcaugacagaacuugggc...........    115        0          seq
    ...........................................ucagugcaugacagaacuugggcc..........    1          0          seq
    ............................................cagugcaugacagaacuugg.............    8          0          seq
    ............................................cagugcaugacagaacuuggg............    2          0          seq
    .............................................agugcaugacagaacuugg.............    5          0          seq
    .............................................agugcaugacagaacuugggc...........    1          0          seq
```
